# Progress in Surgery

**Published:** 1897-07-17

**Authors:** 


### PROGRESS IN SURGERY.

### RHINOLOGY.

(*Continued from page* 252.)

Another interesting and noteworthy remark of Tilley's deserves mention, viz., that if the case is one due to nasal disease, possibly the middle meatus will be filled with granulations, or several large polypi may be present. On the outer wall of the middle meatus in a long-standing case one may often see a rounded swelling of the mucous membrane on a broad base; it does not contain pus, and he says he cannot explain its origin unless it is due to irritation of pus, and is a hyperplasia; but he has often seen it, and when it is present it is pathognomonic of antral suppuration. It is often mistaken for cleavage of the middle turbinate. Tilley has seen it in a gentleman whose nostril never contained a drop of pus, but when he eventually explored his antrum Tilley was surprised to find it full of foul pus. He had drained his left antrum, where there were unmistakable signs of suppuration, and his sensations in the right made him positive of his diagnosis; but he never saw pus in the meatus, though he examined it at least six times.

On the question of treatment Tilley refers to the canine fossa operation thus: "The mucous membrane over the anterior ant[illegible] wall under the lip is divided, and the periosteum t[illegible]ned off, and a plate of bone the size of a sixpence removed; the cavity is then well curetted, and sometimes the mucous membrane actually dissected out, and a counter-opening made in the inferior meatus. The antrum is now packed for forty-eight hours with iodoform gauze, and then syringing of lotions is adopted. Sometimes a good result ensues; but at others we have to reckon with facial œdema and pain for many hours, and sometimes days, after operation, malformation of a sunken cheek, the difficulty of at first keeping open the aperture in the canine fossa, or may be its non-closure, to say nothing of a return to free suppuration. I have nothing to say against the operation in necessary cases; it has been successful in some cases where other means failed, besides which it is founded on a scientific basis, and one can imagine cases where it may be as necessary as it sometimes is to open an attic or antrum mastoideum in chronic aural discharges. I do maintain, however, that it is an operation which should only be done as a last resort, when the patient insists on 'anything to cure,' and when you have explained to him the risks and uncertain result of the proceeding. In these cases the wise surgeon is often he who knows when to stay his hand." We quote these remarks, in which we fully concur, as the severe operation is often needlessly resorted to in our opinion.

**Frontal Sinus.**—An article by Quarry Silcock[8] on distension by mucus and empyema of the frontal sinus affords much valuable information. He remarks that males are more frequently affected than females, probably by reason of the fact that the former are more exposed to injury than the latter. Inasmuch as the frontal sinus is practically undeveloped until the age of seven, it follows that distension could not occur until after that age, and, as would naturally be expected, is more frequent in young and middle-aged than in old people. The symptoms of mucous accumulation vary much in intensity. When the obstruction is acute, as may be the case in rhinitis, the pain is considerable, sometimes throbbing in character, and referred to the frontal region; there may then be tenderness on pressure over the anterior wall of the sinus. In more chronic cases there may be little pain, or at most a feeling of distension over the brows. As the mucus accumulates so does the affected sinus suffer expansion, with the well recognised symptoms and physical signs, the distension being most marked in the direction of the orbit, the frontal sinus wall being here the thinnest and least resistant. The swelling resulting here may be inelastic to touch, so that it may be set down as a bony or cartilaginous tumour; when pressure is made upon it, however, its thin, bony wall will generally give a little. Should the bone have been more completely absorbed, a circumscribed, fluctuating swelling may be apparent, which may be mistaken for a dermoid cyst; the latter, however, is, of course, congenital in origin. Unless the obstruction is complete and osseous, suppuration is a probable complication, for the sinus containing stagnant mucus being in direct communication with the nose, infective organisms will readily make their way into it. Empyema of the frontal sinus is not so often met with as simple mucous distension, and the pus is generally mixed with the retained mucus. When suppuration has occurred, inflammatory symptoms are apt to manifest themselves in fever, more acute tenderness over the anterior wall of the sinus, with perhaps œdema and hyperæmia of the upper lid and brow of the affected side. If the infundibulum is patent, pus or muco-pus may be seen trickling over the inferior turbinated bone. Should the passage be blocked, the abscess eventually breaks through the skin. As possible complications Silcock mentions perforation or necrosis of the posterior wall with consequent suppurative meningitis, orbital cellulitis, absorption of the septum, and maxillary empyema.

Tilley[9] remarks that if, on washing out the antrum, no pus returns with the lotion, and there is still a quantity in the middle meatus, we may be fairly sure that the ethmoidal cells or frontal sinuses are at fault. He then advises to take a probe (Hajek's) and try to pass it very carefully into the fronto-nasal canal; remembering that this is beyond the anterior end of the middle turbinate and on the outer side of the meatus. If one can pass it into the frontal sinus, the patient can generally feel it is there, and its withdrawal is followed by a free flow of pus. He utters a warning against the use of any force.

Silcock[10] describes his method of operating thus: "An anæsthetic is administered, and an incision about one inch in length is made below and under cover of the eyebrow over the most prominent part of the swelling if there be one, and in any case down to the bone; or if the bone be absorbed the cavity of the sinus is opened at once. The opening through the floor of the sinus is enlarged with a gouge until it is of sufficient size to allow of the insertion of the tip of the finger, and then a sharply-curved eyed probe is passed into the infundibulum, and if it be sufficiently curved its end should appear at the corresponding anterior nares. A steel probe, devised by M. Panas, is a very convenient form. A silver wire or strong silk thread is now drawn through the track of the probe, and by means of one or the other a thick leaden wire is dragged through the infundibulum into the nose. I have used a soft French aseptic catheter with advantage instead of a probe; by its means the silk thread can be drawn through the nose into the wound. The ends of the leaden wire may be hooked over the ala of the nose and the edges of the wound, so avoiding any danger of its slipping in or out. If the infundibulum is obliterated by bone, a new passage must be forced by means of a bradawl or sharp steel probe; in such circumstances the tip of a finger should be thrust inside the anterior nares as a guide to the direction of the probe. Instead of one strand of leaden wire, three or four may be employed; the wire should be retained in position until the discharge ceases to be purulent, the sinus being irrigated by some antiseptic solution twice or thrice daily, when some of the fluid should find its way out by the nose. On removing the wire drain the sinus and infundibulum may be washed out by means of a lachrymal syringe fitted with a sufficiently long and suitably bent nozzle. If the wound fails to heal, a fistulous tract remaining, it must be assumed that the drain into the nose is ineffective, and the passage must be restored. Should the mucous membrane lining the sinus be hypertrophic or the seat of polpi, the curette must be freely employed."

[7] Lancet, Sept. 26, 1896. [8] Practitioner, March, 1897. [9] Loc. cit [10] Loc. cit.

